# Supplementary material for: A gradient oxy-thiophosphate-coated Ni-rich layered oxide cathode for stable all-solid-state Li-ion batteries
Source: Nat Commun. 2023 Jan 10;14:146. doi: 10.1038/s41467-022-35667-7 (PMC9832028; doi:10.1038/s41467-022-35667-7)
Supplement: Supplementary file 1 — Supplementary Information [file 41467_2022_35667_MOESM1_ESM.pdf]

Supplementary Information

A gradient oxy-thiophosphate-coated Ni-rich layered oxide cathode for stable all-solid-state Li-ion batteries

Jianwen Liang,[1+] Yuanmin Zhu,[2,3+] Xiaona Li,[1+] Jing Luo,[1] Sixu Deng,[1] Yang Zhao,[1] Yipeng Sun,[1] Duojie Wu[3] Yongfeng Hu,[4] Weihan Li,[1,5] Tsun-Kong Sham,[5] Ruying Li,[1] Meng Gu,[3] Xueliang Sun[1]**

[1] Department of Mechanical and Materials Engineering, University of Western Ontario, London, Ontario N6A 5B9, Canada.

[2] School of Material Science and Engineering, Dongguan university of technology, Dongguan, Guangzhou, China

[3] Department of Materials Science and Engineering, Southern University of Science and Technology, Shenzhen, 518055, China.

[4] Canadian Light Source, 44 Innovation Boulevard, Saskatoon, SK, S7N 2V3, Canada

[5] Department of Chemistry, University of Western Ontario, London, ON, N6A 5B9, Canada

* Corresponding email: gum@sustech.edu.cn; xsun9@uwo.ca

[+] These authors have contributed equally.

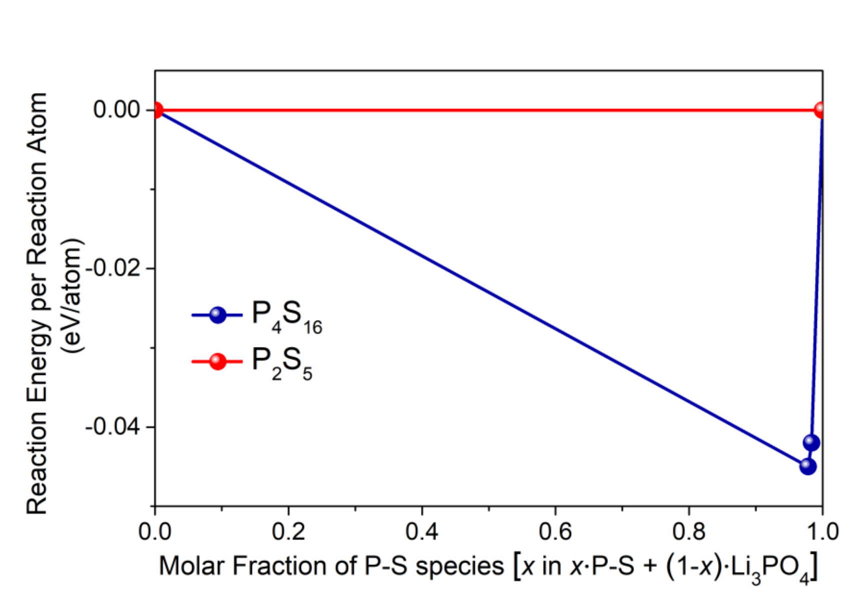

Supplementary Figure 1. The reaction energy between P-S species and Li_3PO_4. Red, the reaction between P_2S_5 and Li_3PO_4; bule, the reaction between P_4S_{16} and Li_3PO_4.

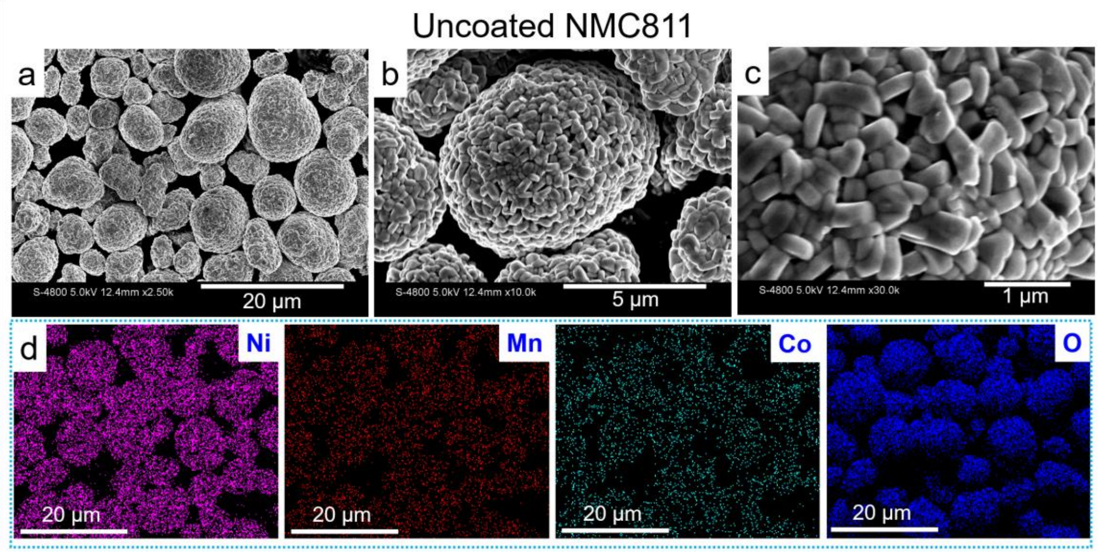

Supplementary Figure 2. Morphology of uncoated NMC811 powder. (a-c) SEM images at different magnifications and (d) EDX mapping for Ni, Mn, Co, and O elements.

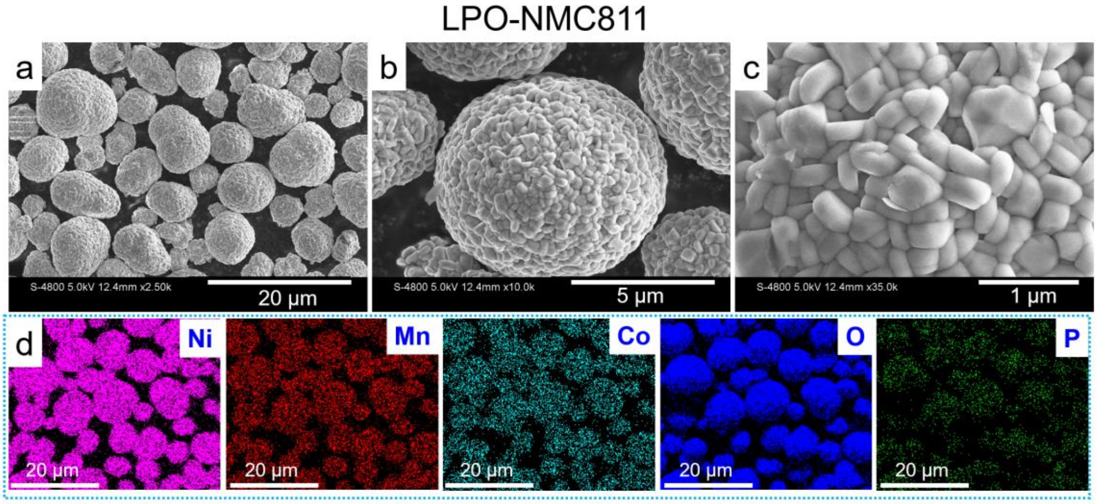

Supplementary Figure 3. Morphology of LPO-NMC811 powder. (a-c) SEM images at different magnifications and (d) EDX mapping for Ni, Mn, Co, O, and P elements.

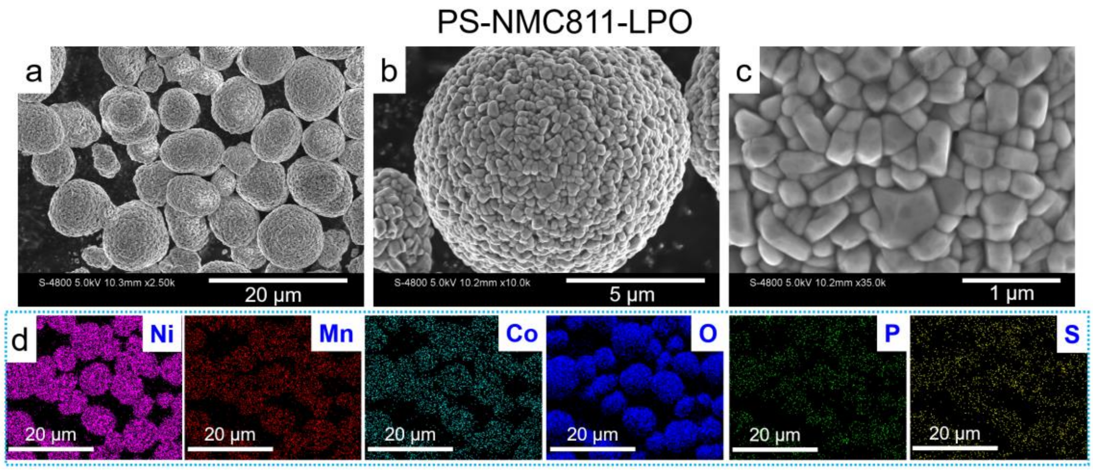

Supplementary Figure 4. Morphology of PS-LPO-NMC811 powder with 1wt% P_4S_{16} content. (a-c) SEM images at different magnifications and (d) EDX mapping for Ni, Mn, Co, O, P, and S elements.

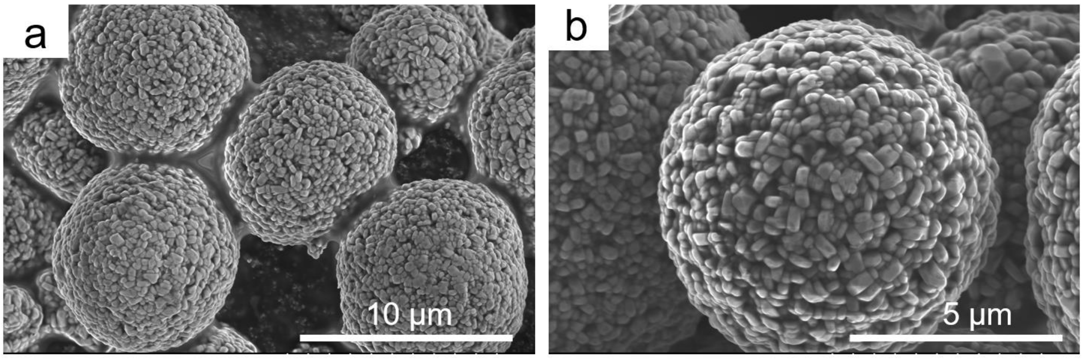

Supplementary Figure 5. Morphology of PS-LPO-NMC811 powder with 5wt% P_4S_{16} content.

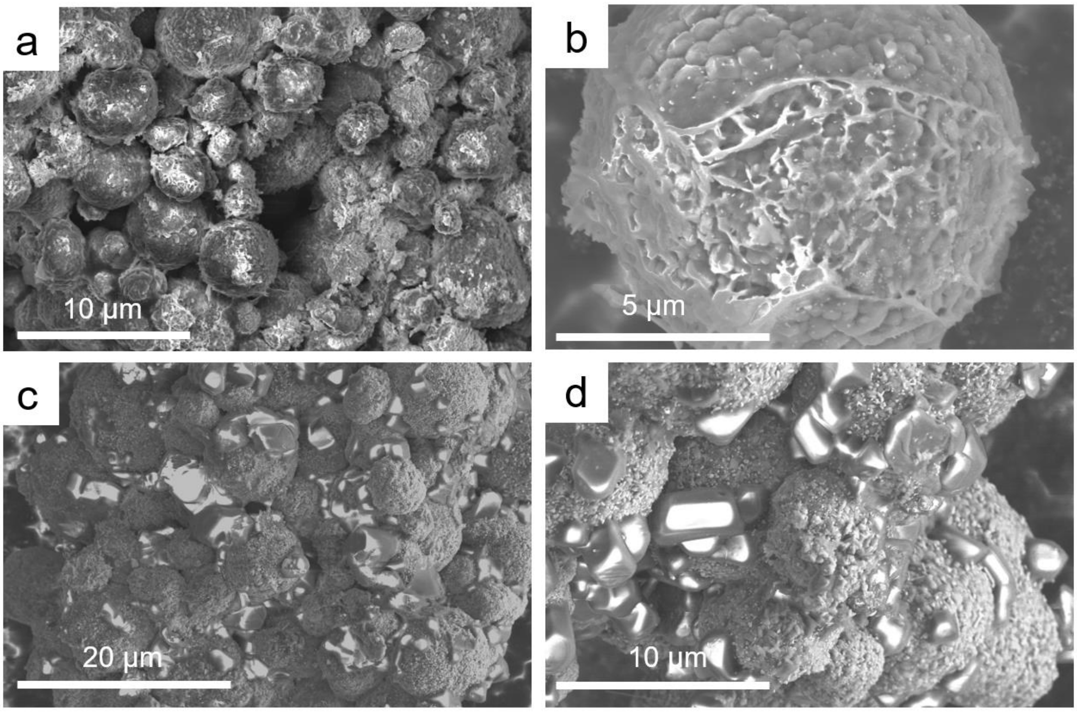

Supplementary Figure 6. Morphology of PS-LPO-NMC811 powder (5 wt.% P_4S_{16}) after anneal at (a,b) 350 °C and (c,d) 450 °C.

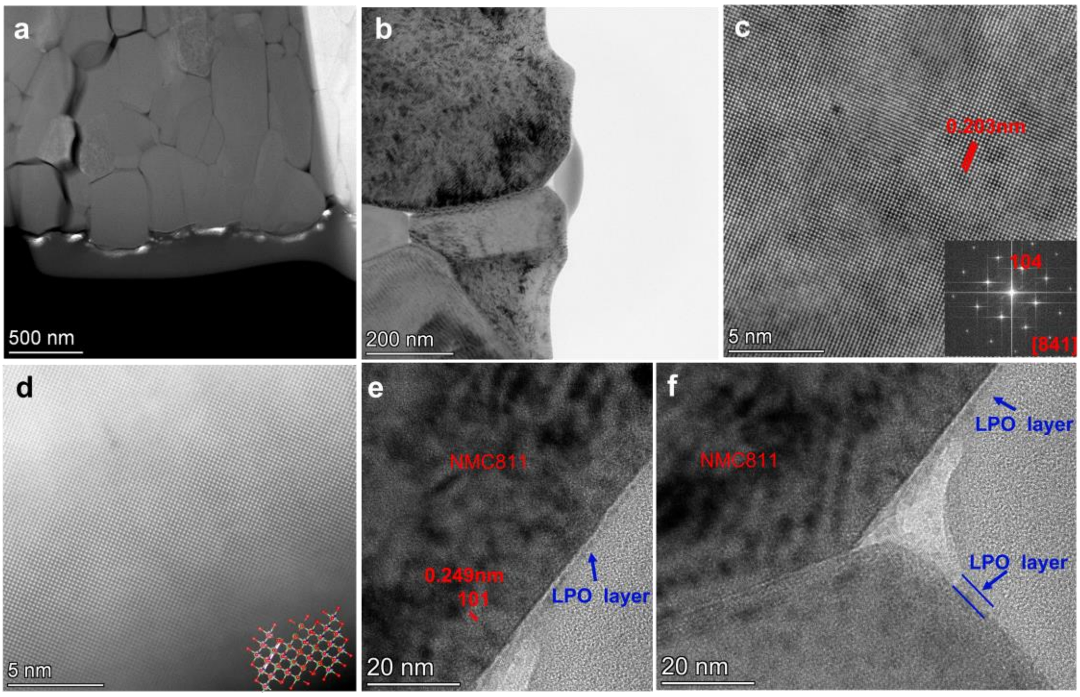

Supplementary Figure 7. The (a,b) cross-sectional HRSTEM and (c-f) HRTEM images of the LPO-NMC811 powder, the clear cross-section was cut by focused ion beam after the ALD coating process.

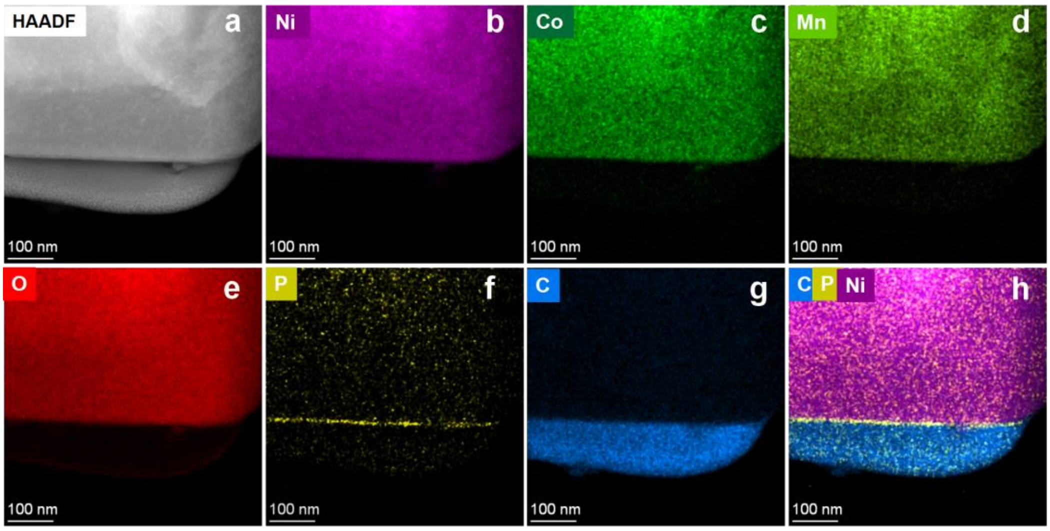

Supplementary Figure 8. (a) The HAADF-STEM image of the LPO-NMC811 powder, (b-g) the corresponding EDX elemental mapping of Ni, Co, Mn, O, P, C, and (h) the overlay map of these elements.

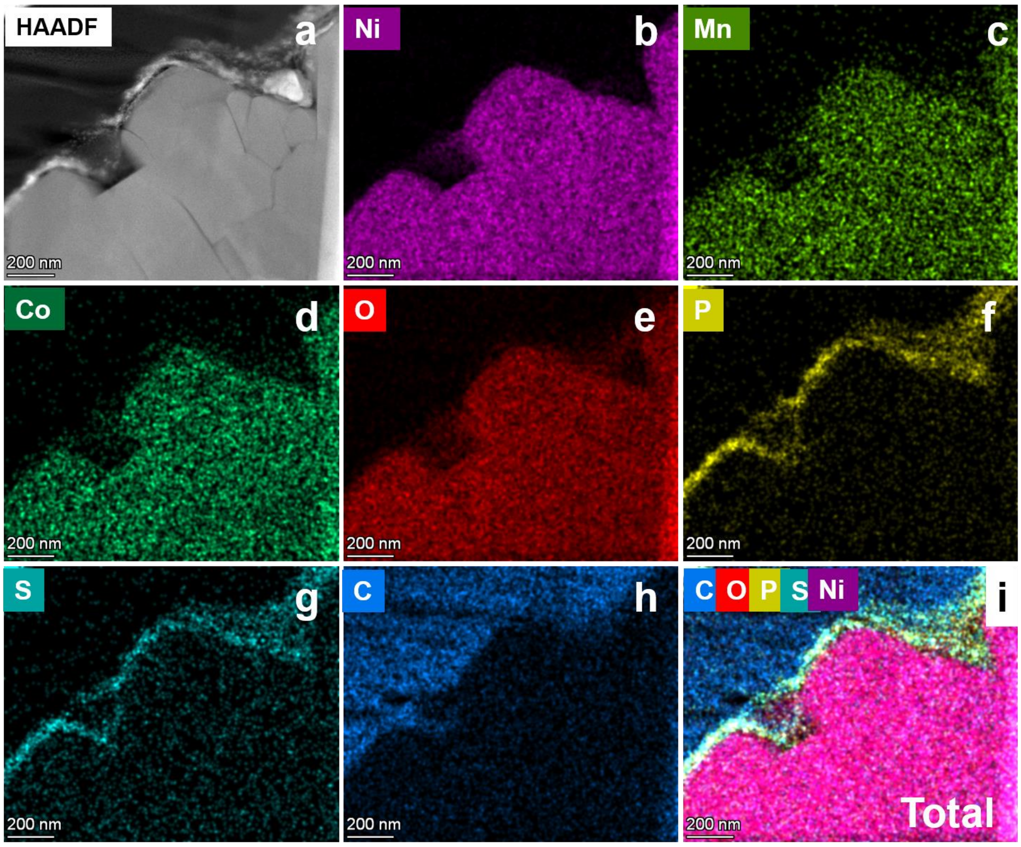

Supplementary Figure 9. (a) The STEM-HAADF image of the PS-LPO-NMC811 powder section cut by focused ion beam, (b-h) the corresponding EDX elemental maps of Ni, Mn, Co, O, P, S, C, and (i) their overlay map.

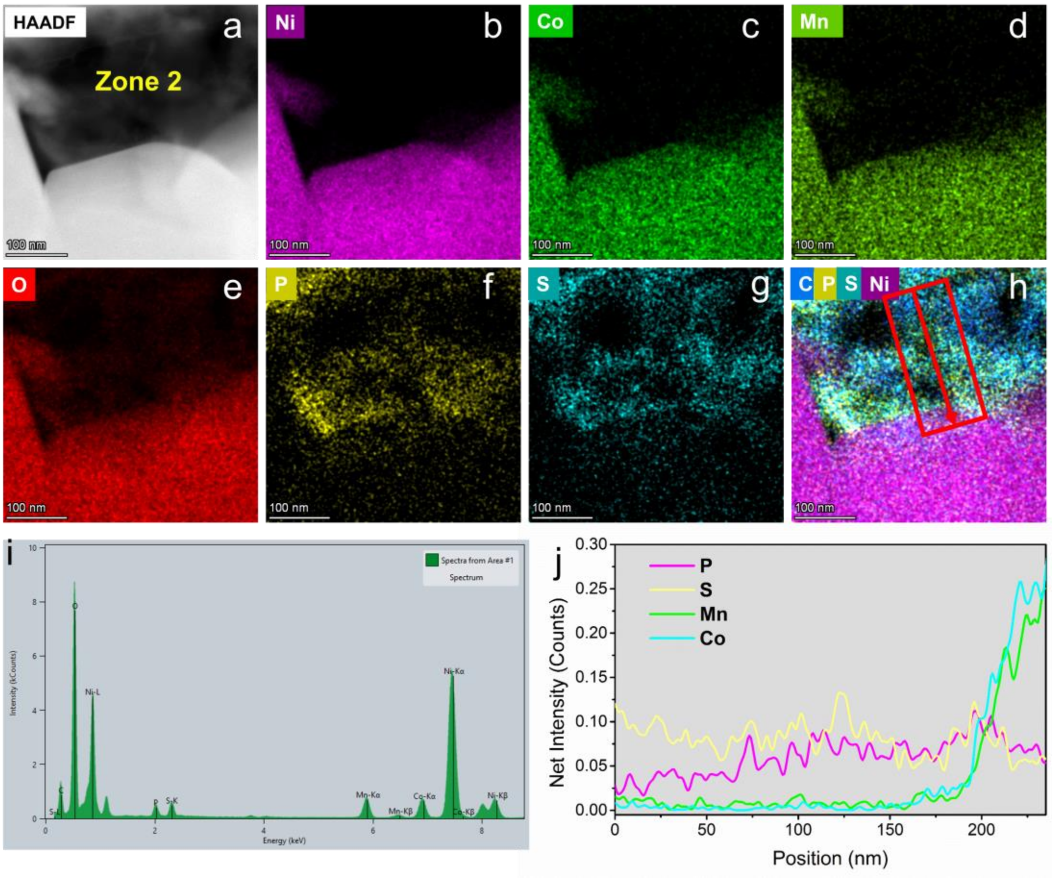

Supplementary Figure 10. (a) STEM-HAADF image of the inner primary particles (Zone 2 in figure 2a) of PS-LPO-NMC811 powder, and (b-h) the corresponding EDX elemental maps of Ni, P, S, and their overlay map. (i-j) An EDS spectrum and EDS line scan (from figure h) of the inner primary particles marked in figure h.

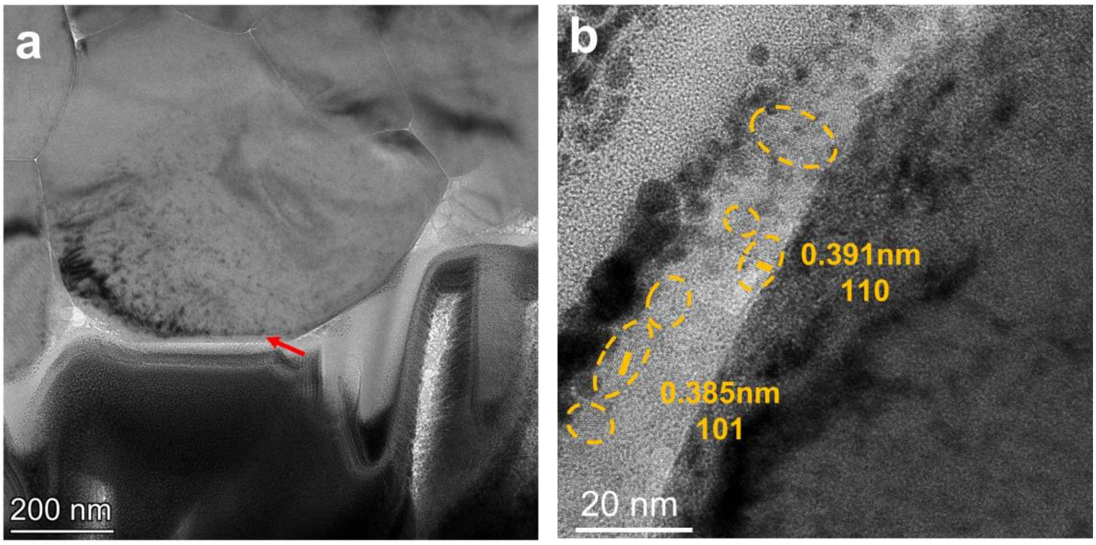

Supplementary Figure 11. HRTEM images showing the morphology of the $Li_3P_{1+x}O_4S_{4x}$ coating on the NMC811 particles for the PS-LPO-NMC811 powder sample.

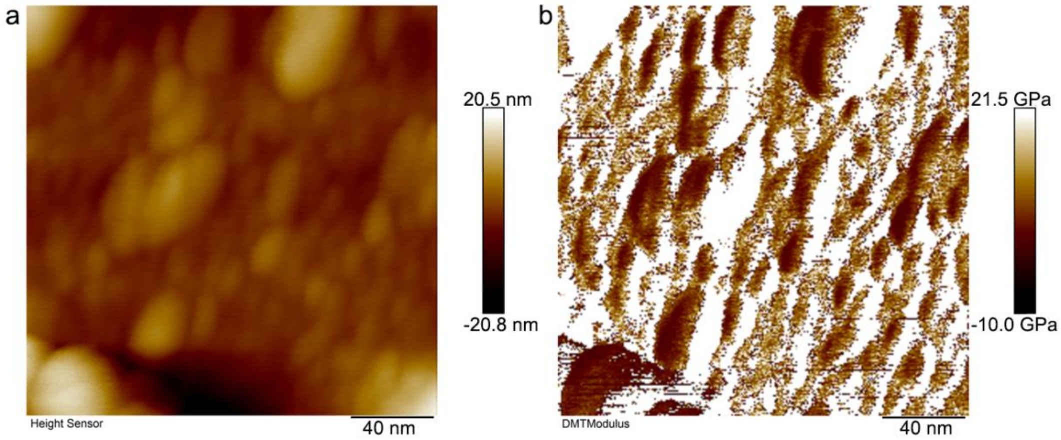

Supplementary Figure 12. (a) Atomic force microscopy (AFM) image and (b) corresponding DMT modulus mapping of the PS-LPO-NMC811 powder sample.

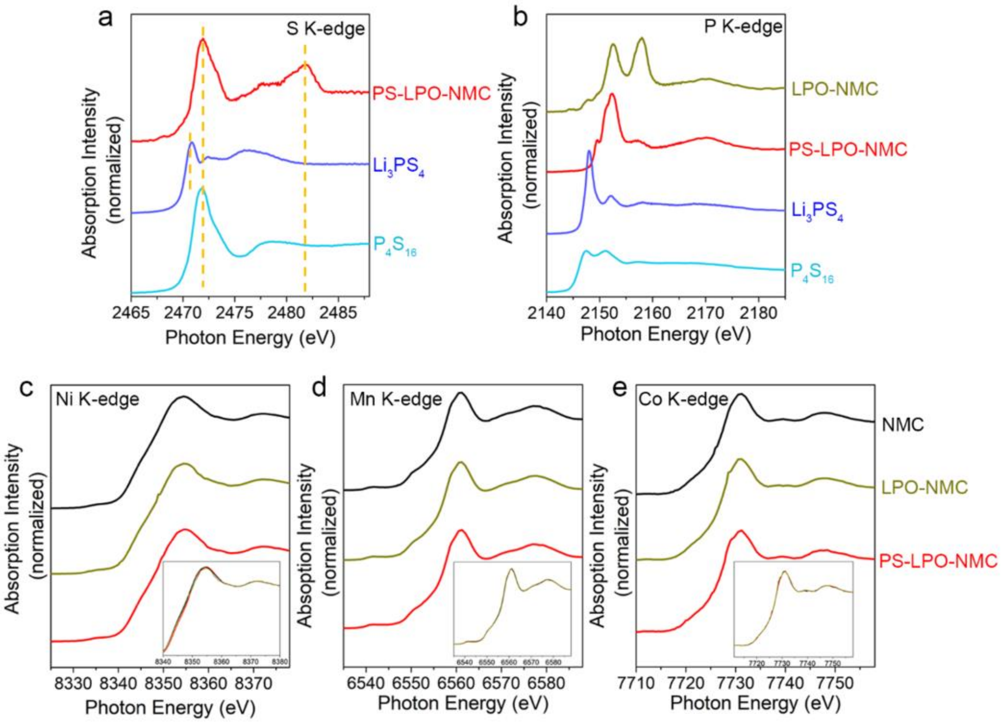

Supplementary Figure 13. XANES spectra of NMC, LPO-NMC, and PS-LPO-NMC powder samples. (a) S K-edge XANES spectra of P_4S_{16} (cyan), commercial Li_3PS_4 (blue), and PS-LPO-NMC (red), (b) P K-edge XANES spectra of P_4S_{16} (cyan), commercial Li_3PS_4 (blue), LPO-NMC (brown), and PS-LPO-NMC (red). (c) Ni K-edge, (d) Mn K-edge, and (e) Co K-edge XANES spectra of bare NMC (dark), LPO-NMC (brown), and PS-LPO-NMC (red).

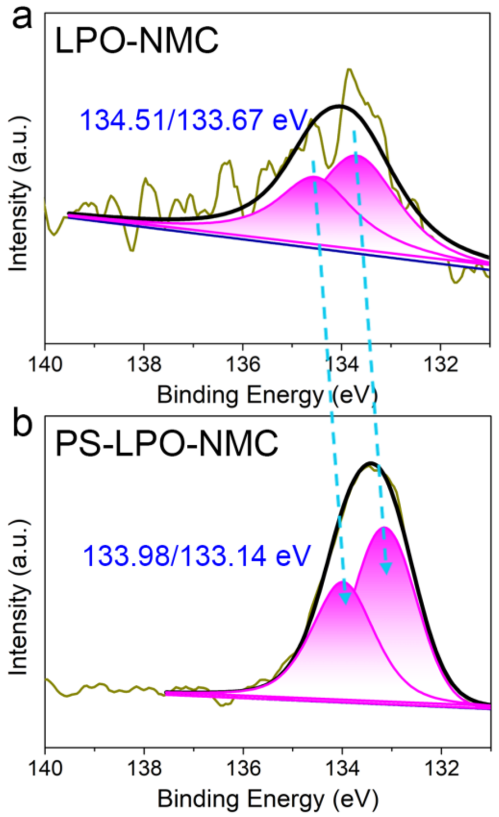

Supplementary Figure 14. P 2*p* XPS spectra of (a) LPO-NMC811 and (b) PS-LPO-NMC811 powder samples.

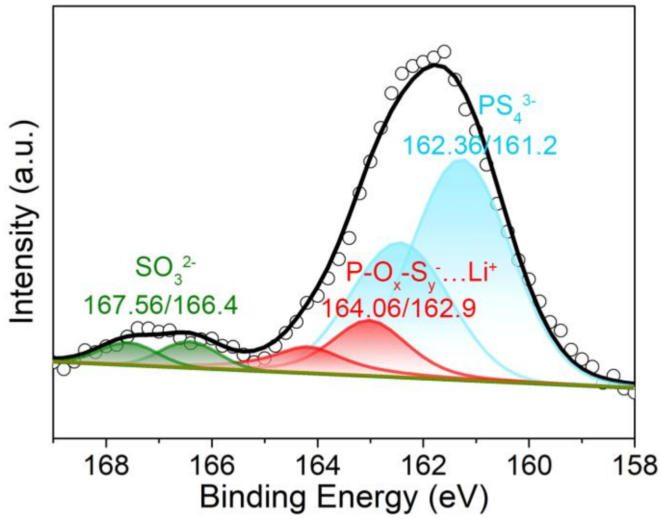

Supplementary Figure 15. S 2*p* XPS spectra of PS-LPO-NMC811 powder sample.

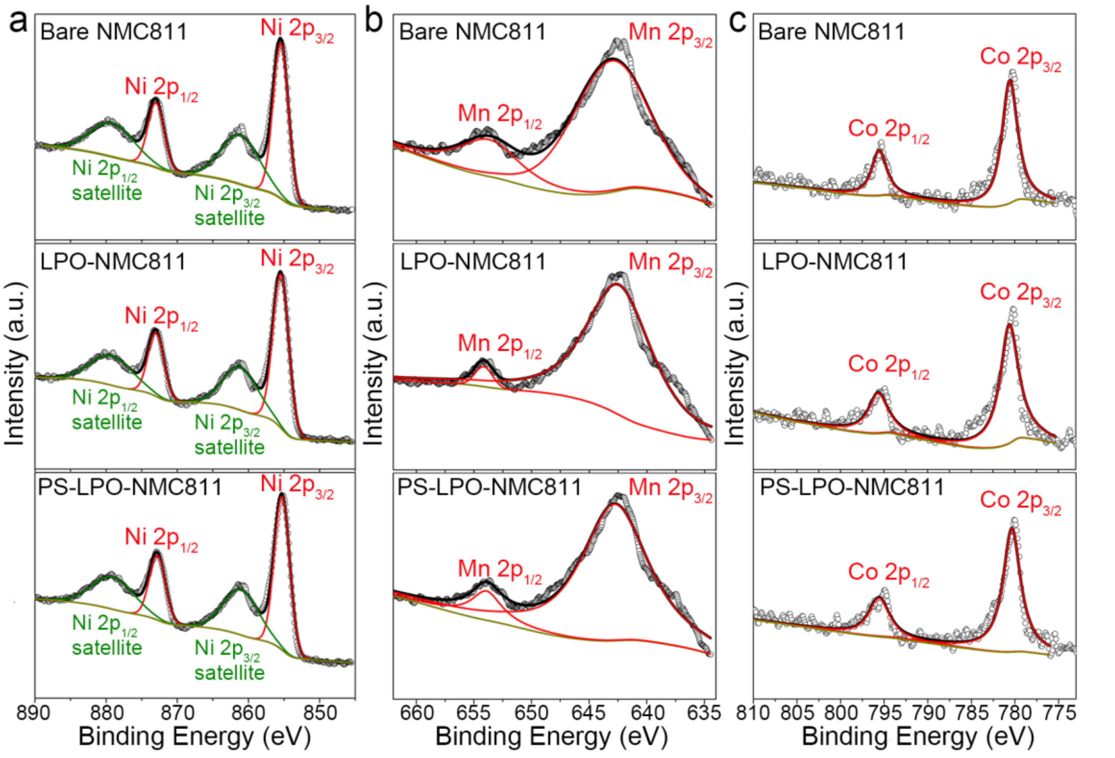

Supplementary Figure 16. The comparison of (a) Ni 2*p*, (b) Mn 2*p*, and (c) Co 2*p* XPS spectra for bare NMC811, LPO-NMC811, and PS-LPO-NMC811 powder samples.

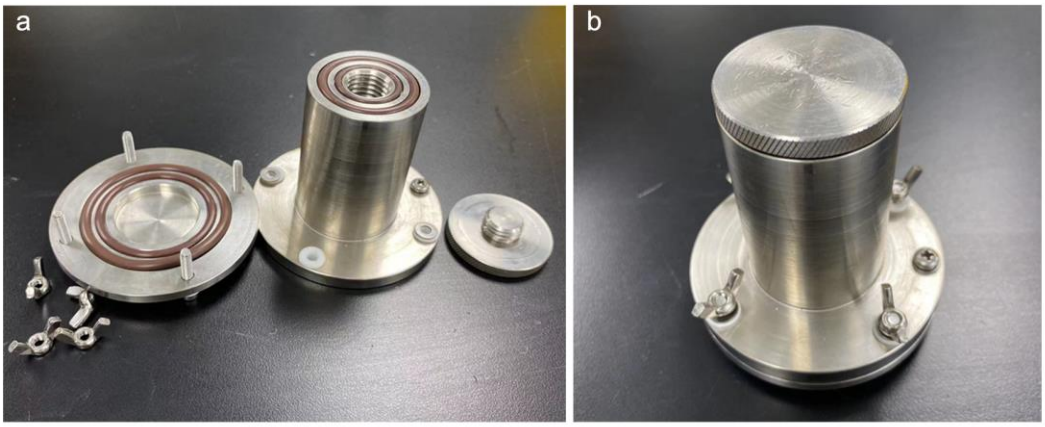

Supplementary Figure 17. (a) A disassembled and (b) assembled homemade KP-Solid cells.

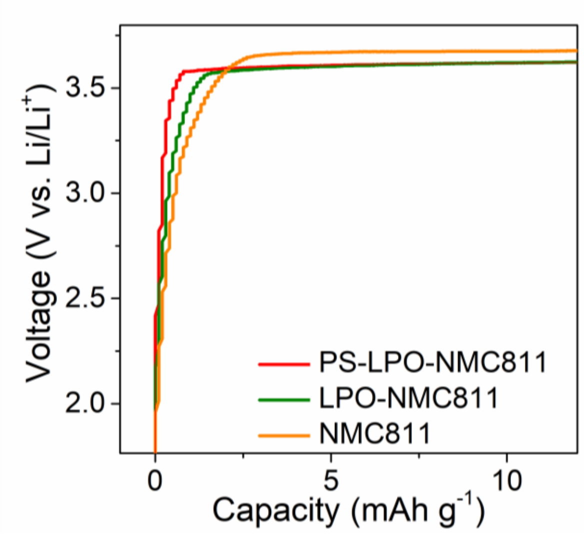

Supplementary Figure 18. The beginning of the charging process of the three In|LGPS|NMC811 (orange), In|LGPS|LPO-NMC811 (green), and In|LGPS|PS-LPO-NMC811 (red) cells in the first cycle at the current density of 0.089 mA cm^{-2} at 25℃.

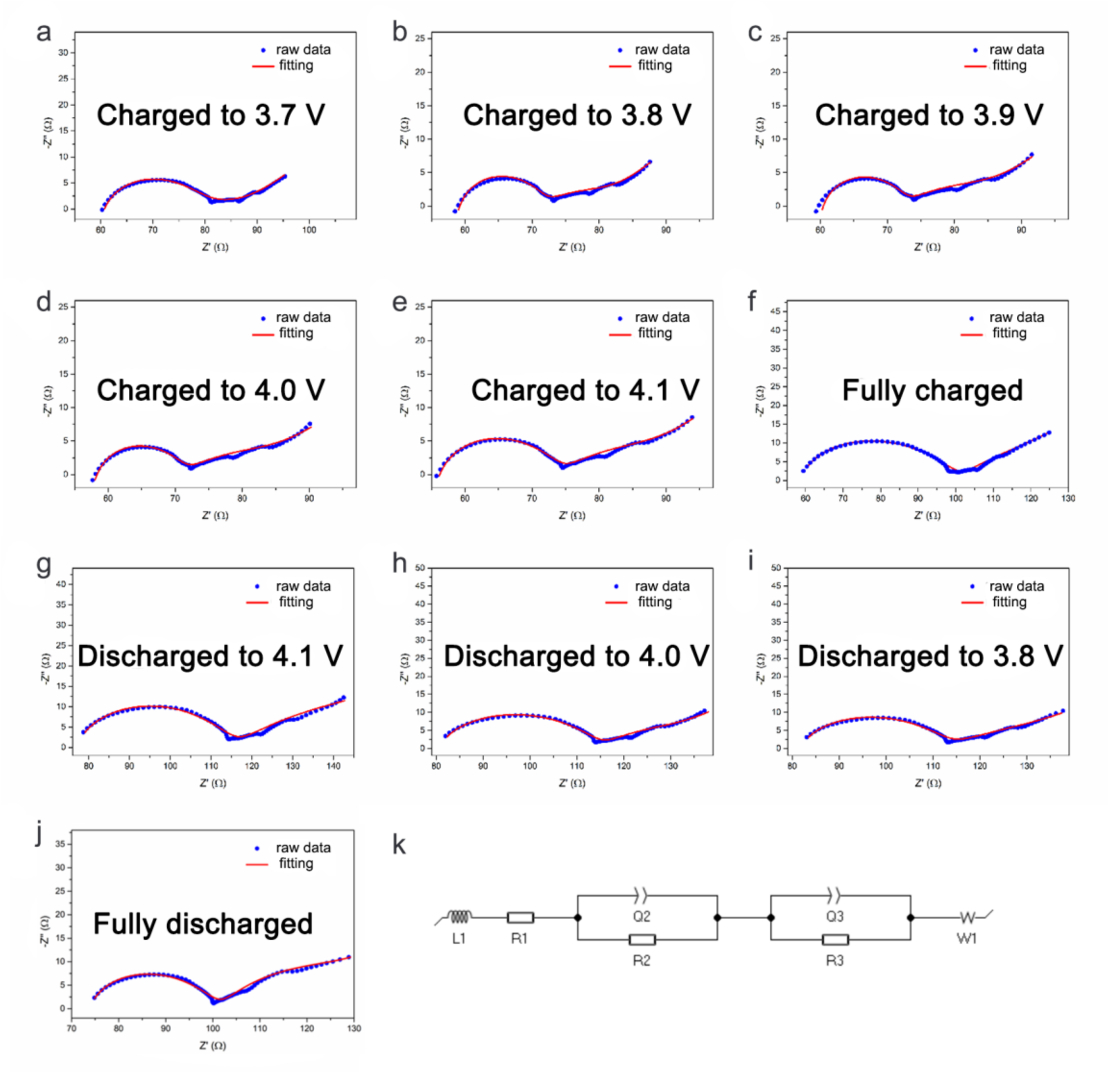

Supplementary Figure 19. Electrochemical impedance spectroscopy measurements of In|LGPS|PS-LPO-NMC811 cell cycled at 0.178 mA cm^{-2} at different charge/discharged states. (a) Charged to 3.7 V, (b) charged to 3.8 V, (c) charged to 3.9 V, (d) charged to 4.0 V, (e) charged to 4.1 V, (f) fully charged, (g) discharged to 4.1 V, (h) discharged to 4.0 V, (i) discharged to 3.8 V, and (j) fully discharged. (k) The equivalent circuit.

The electrochemical impedance spectra were analyzed by EC-lab V11.02 software [1]. The raw and fitted curves are shown in Supplementary Figure 19a-j with an equivalent circuit of L1+R1+CPE2//R2+CPE3//R3+W1 (Supplementary Figure 19k) [2-5]. The L1 is electrical inductance related to the external circuit. The R1 corresponds to the resistance of the electrolyte. CPE2//R2 is assigned to the capacitance and resistance of the interface between the cathode and electrolyte in the high-frequency region. The capacitance and resistance of the interface between the anode and electrolyte in the middle-frequency region can be modeled using CPE3//R3. The Warburg

impedance (W1) represents the diffusion of the ions within the cathode. Fitted values of each element in different voltage states during the charge/discharge process regarding the spectra in Supplementary Figure 19 are collected as in Supplementary Table 3. The impedance of electrolyte (R1) reveals similar data with 55±3.8 Ω during the charging process and ~74±4.5 Ω during discharging process. A stable capacitance and resistance of the interface between the cathode and electrolyte (C2 and R2) can be revealed. Moreover, the capacity of the interface is low as 10^{-6} F. The resistance of the interface between the anode and electrolyte shows a small change from 7 Ω in the initial state to 49.83 Ω in the full charge state and then returns back to 31.58 Ω in the full discharge state.

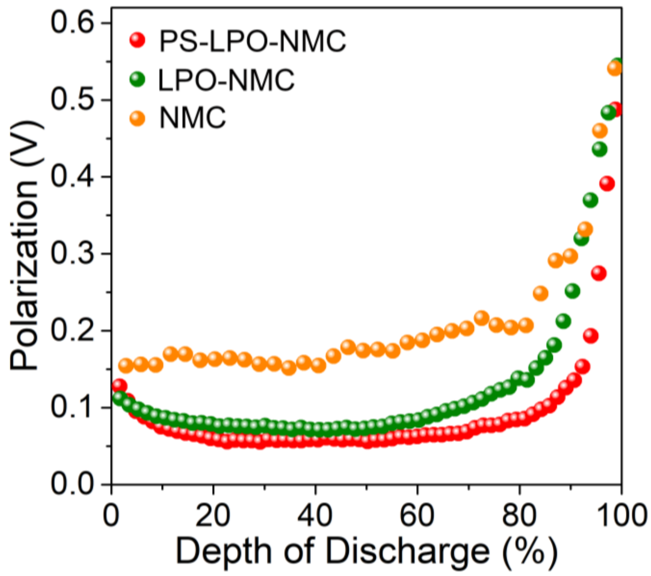

Supplementary Figure 20. The polarization voltages of the three In|LGPS|NMC811 (orange), In|LGPS|LPO-NMC811 (green), and In|LGPS|PS-LPO-NMC811 (red) cells at different discharge states during the GITT test in Figure 5 (b,c). The error bar is around 2%, which is original from the mechanism error bar in this testing (±0.5 μA) and the data analysis (±0.1 mV).

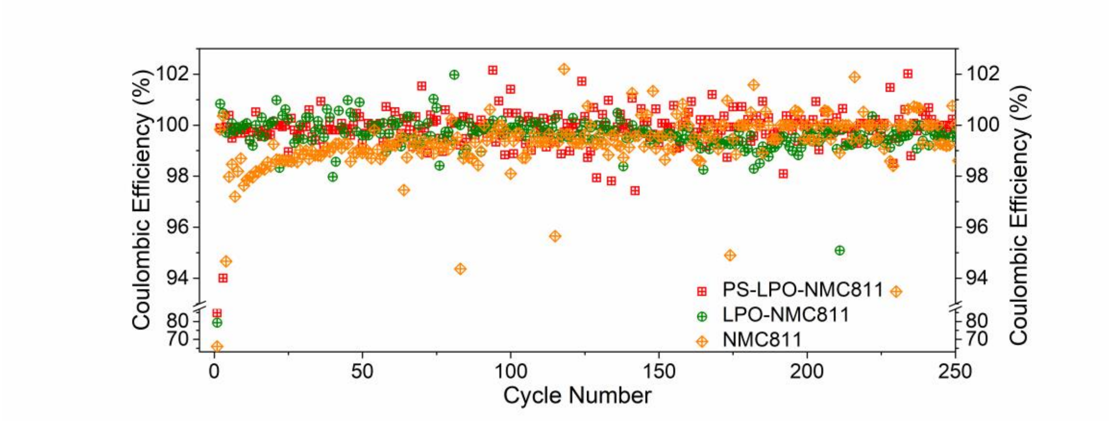

Supplementary Figure 21. The Coulombic efficiencies of the three In|LGPS|NMC811 (orange), In|LGPS|LPO-NMC811 (green), and In|LGPS|PS-LPO-NMC811 (red) cells for the long cycling test at 25 ℃ in Figure 5h. The current is 0.089 mA cm^{-2} for the initial three cycles and 0.178 mA cm^{-2} for the following cycles.

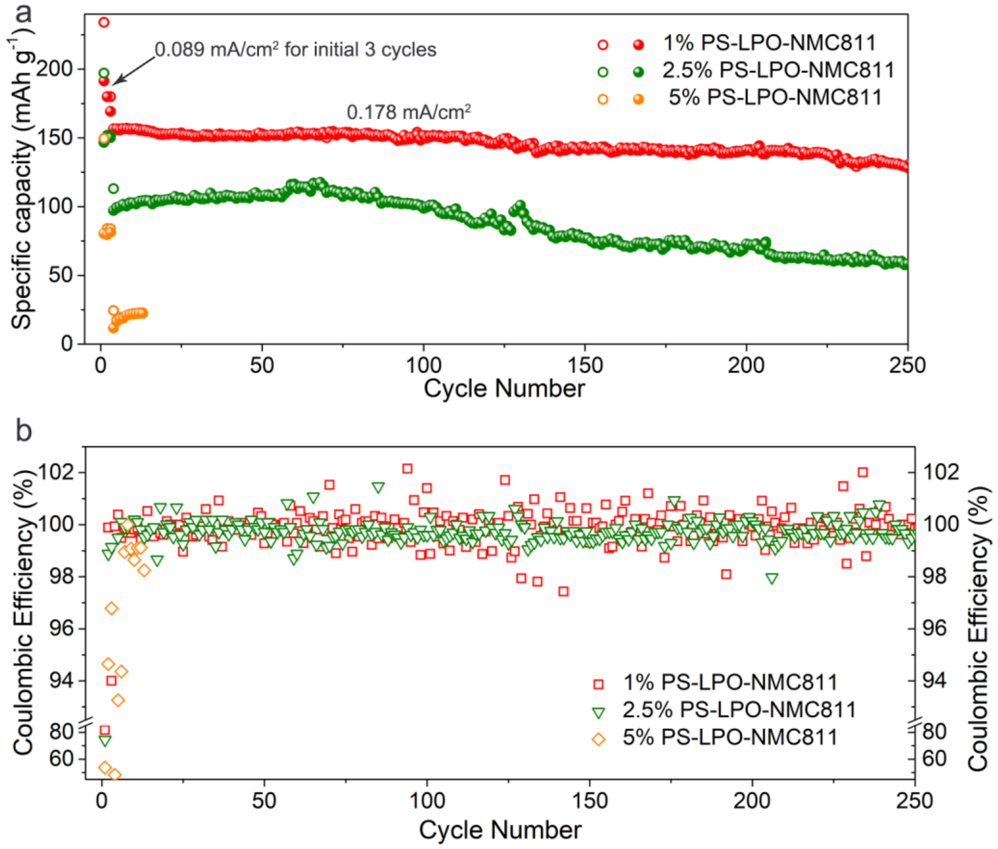

Supplementary Figure 22. The (a) cycling performance and (b) corresponding Coulombic efficiencies of different PS-LPO-NMC811 cathodes prepared by different weight fractions of P_4S_{16}

in the sulfurization process at 25 °C. The three cells are In|LGPS|1%PS-LPO-NMC811, In|LGPS|2.5%PS-LPO-NMC811, and In|LGPS|5%PS-LPO-NMC811. The current is 0.089 mA cm^{-2} for the initial three cycles and 0.178 mA cm^{-2} for the following cycles.

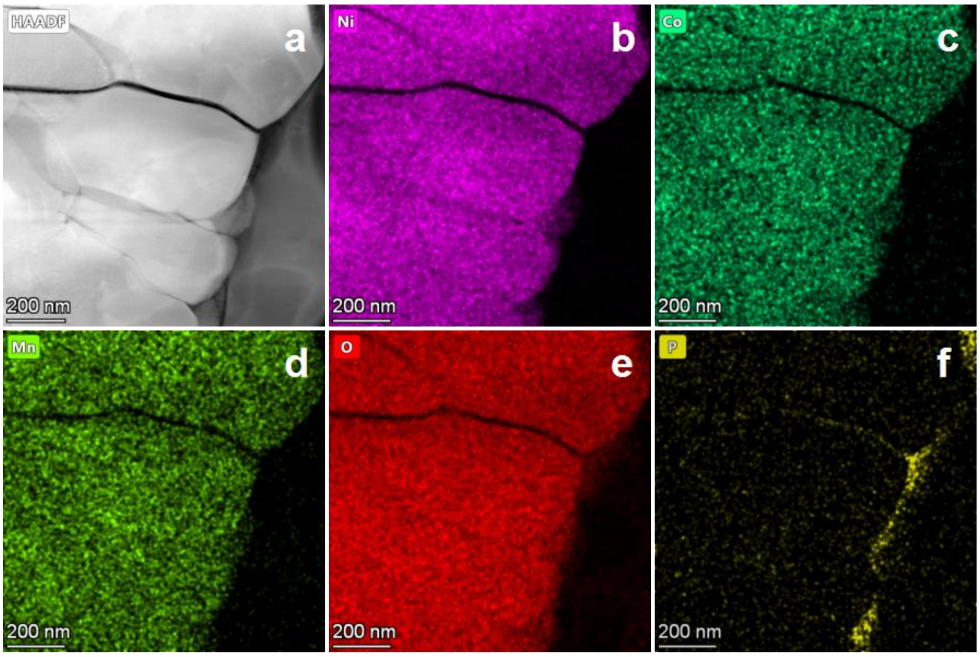

Supplementary Figure 23. A STEM-HAADF image of the surface of LPO-NMC811 particles after 100 cycles at the fully discharged state, the corresponding EDX elemental map, and the overlay map of these elements in the sample. Note that the TEM measurements are ex situ postmortem positive electrode measurements. The In|LGPS|LPO-NMC811 cell was cycled at 0.089 mA cm^{-2} for the initial three cycles and 0.178 mA cm^{-2} for the following cycles at 25 °C.

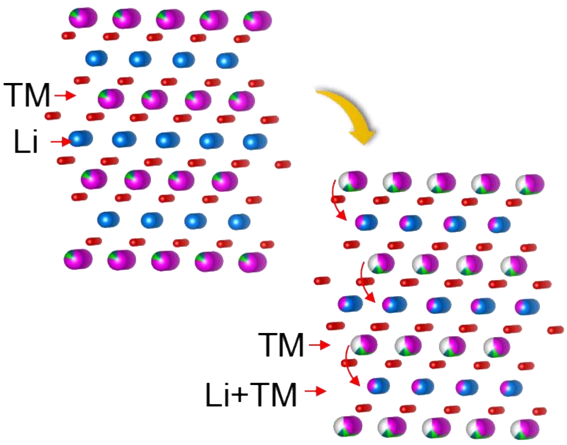

Supplementary Figure 24. Schematic representation of the possible mechanism of layered to rock-salt transformation.

Supplementary Table 1. S 1s HEXPS spectra of the PS-LPO-NMC811 at different photon energies.

**Photon Energy (eV)**	**S 1s**		
	**thiophosphate species ($P\text{-}S^{-}...Li^{+}$)**	**oxy-thiophosphate species ($P\text{-}O_x\text{-}S_y^{-}...Li^{+}$)**	**Intensity ratio of thiophosphate to oxy-thiophosphate species**
	Position (eV)	Position (eV)	(%)
**3000**	2468.4(4)	2470.2(2)	91.9(3)
**6000**	2468.5(9)	2470.2(9)	113.3(9)
**8000**	2468.7(0)	2470.5(2)	167.9(1)

Supplementary Table 2. O 1s HEXPS spectra of the PS-LPO-NMC811 at different photon energies.

**Photon Energy (eV)**	**O 1s**		
	**LPO**	**oxy-thiophosphate species ($P\text{-}O_x\text{-}S_y^{-}...Li^{+}$)**	**Intensity ratio of LPO to oxy-thiophosphate species**
	Position (eV)	Position (eV)	-
**3000**	531.5(0)	533.1(8)	2.1(4)
**6000**	531.5(0)	533.2(1)	2.5(7)
**8000**	531.5(0)	533.2(2)	3.3(7)

Supplementary Table 3. The simulated parameters for the EIS of the In|LGPS|PS-LPO-NMC811 cell cycled at 0.178 mA cm^{-2} at 25 °C at different charge/discharged states..

States (vs. Li^{+}/Li)	L1/H	R1/Ω	C2/F	R2/Ω	C3/F	R3/Ω	s1/ $\Omega S^{-1/2}$	$\chi^2/\lvert Z\rvert$
Charged to 3.7 V	2.389e-6	57.35	1.1216e-6	25.27	0.03531	7.037	1.518	4.654e-3
Charged to 3.8 V	5.52e-6	57.39	1.17e-6	14.07	0.01171	11.99	1.404	1.552e-3
Charged to 3.9 V	6.429e-6	55.82	1.032e-6	15.3	0.03038	16.98	1.346	0.04244
Charged to 4.0 V	8.007e-6	53.74	0.6792e-6	18.56	0.02495	12.73	1.589	1.461e-3
Charged to 4.1 V	6.492e-6	53.11	0.8963e-6	20.62	0.052 93	18.72	1.511	1.624e-3
Fully charged	7.649e-6	51.27	0.281 7	25.54	0.562 2	49.83	2.02	2.052e-3
Discharged to 4.1 V	5.026e-6	72.39	0.7157e-6	33.31	0.385 7	48.67	0.8972	2.402e-3
Discharged to 4.0 V	4.17e-6	76.78	0.7839e-6	35.11	0.073 6	23.97	1.864	2.443e-3
Discharged to 3.8 V	4.186e-6	78.52	0.8174e-6	34.46	0.067 54	23.9	1.773	1.745e-3
Fully discharged	5.552e-6	70.13	0.7537e-6	30.85	0.097 19	31.58	1.396	1.947e-3

References:

1. Alavi S. M. M., Birkl C. R., Howey D. A., *J. Power Sources*, 2015, 345-352.
2. L. Yuan, X. Qiu, L. Chen, and W. Zhu, J. Power Sources, 2009, 189, 127. 2. Vadhva, P., Hu, J., Johnson, M. J., Stocker, R., Braglia, M., Brett, D. J., & Rettie, A. *J. ChemElectroChem* 2021, 8, 1930-1947.
3. Chen, K., Yamamoto, K., Orikasa, Y., Uchiyama, T., Ito, Y., Yubuchi, S., Hayashi, A., Tatsumisago, M., Nitta, K., Uruga, T. and Uchimoto, Y. S*olid State Ionics* 2018, 327, 150-156.
4. Deysher, G., Chen, Y.T., Sayahpour, B., Lin, S.W.H., Ham, S.Y., Ridley, P., Cronk, A., Wu, E.A., Tan, D.H., Doux, J.M. and Oh, J.A.S. *ACS Appl. Mater. Interfaces* 2022, 14, 47706-47715.
5. Wang, J., Zhang, Z., Han, J., Wang, X., Chen, L., Li, H. and Wu, F. *Nano Energy* 2022, 100, 107528.
